# Effects of a Chinese Patent Medicine Gushen’antai Pills on Ongoing Pregnancy Rate of Hormone Therapy FET Cycles: A Multi-Center, Randomized, Double-Blind, Placebo-Controlled Clinical Trial

**DOI:** 10.3389/fendo.2020.581719

**Published:** 2020-09-23

**Authors:** Xian-ling Cao, Jing-yan Song, Xing-xing Zhang, Yan-hua Chen, Yi-li Teng, Hai-ping Liu, Tai-you Deng, Zhen-gao Sun

**Affiliations:** ^1^ College of Traditional Chinese Medicine, Shandong University of Traditional Chinese Medicine, Jinan, China; ^2^ Integrative Medicine Research Centre of Reproduction and Heredity, The Affiliated Hospital of Shandong University of Traditional Chinese Medicine, Jinan, China; ^3^ Reproductive Medicine Center, Maternity and Child Health Care of ZaoZhuang, ZaoZhuang, China; ^4^ Shanxi Maternal and Child Health Care Hospital, Taiyuan, China; ^5^ The First Affiliated Hospital of Wenzhou Medical University, WenZhou, China; ^6^ The 960th Hospital of the PLA Joint Logistics Support Force, Jinan, China; ^7^ College of Pharmacy, China Pharmaceutical University, Nanjing, China

**Keywords:** randomized controlled trials, frozen-thawed embryo transfer, ongoing pregnancy rate, Gushen’antai pills, hormone therapy

## Abstract

In the past decade, the number of frozen-thawed embryo transfer (FET) has increased dramatically with the expansion of surgical indications and the improvement of freezing related technologies. How to improve the success rate and reduce the adverse effects of FET is our research priorities. This study aimed to investigate the safety and effectiveness of Gushen’antai pills (GSATP) by measuring the ongoing pregnancy rate (OPR) in patients from FET and hormone therapy (HT) cycle. From November 2019 to May 2020, 5 Chinese hospitals conducted a multi-center, randomized, double-blind, placebo-controlled study. In total, 271 HT FET cycles in patients were randomly divided (1:1 ratio) to receive GSATP (6 g, tid) or placebo (6g, tid) for 12 weeks of pregnancy. Patients, clinicians, and researchers were blinded to treatment allocation. The primary endpoint was the OPR at week 12 of pregnancy. The secondary endpoints were vaginal bleeding or brown discharge rate, implantation rate (IR), clinical pregnancy rate (CPR) and abortion rate (AR). Adverse events were recorded during the treatment period. The results showed that the OPR remained higher in the GSATP group when compared to placebo group (56.62% vs. 44.44%, *p* = 0.045). Vaginal bleeding or brown discharge rate was lower in the GSATP group than the placebo group (10% vs. 23.08%, *p* = 0.032), while the IR (35.16% vs. 27.64%, *p* = 0.070), CPR (58.82% vs. 48.15%, *p* = 0.078), incidence of total adverse events (8.09% vs. 3.22%, *p* = 0.051) and AR (3.75% vs. 7.69%, *p* = 0.504) were similar between GSATP and placebo groups. Subgroup analysis showed that there were significant differences in CPR (74.19% vs. 54.17%, *p* = 0.004) and OPR (72.04% vs. 51.04%, *p* = 0.003) between GSATP group and Placebo group when the patient was younger than 35 years old. This multi-center, randomized, double-blinded, placebo-controlled clinical study showed for the first evidence that GSATP may have potential to improve the OPR and decrease vaginal bleeding or brown discharge rate in HT FET cycle patients.

## Introduction

Infertility is an ongoing global reproductive health problem that is characterized by failure to establish a clinical pregnancy after 12 months of regular and unprotected sexual intercourse (1). It is estimated that there are more than 186 million women of child-bearing age who suffer from infertility worldwide, with a ratio of approximately 4:7 in developed and developing countries (2, 3). In vitro fertilization (IVF) is the most common therapeutic choice for treating infertility, and it is performed from almost 40 years since the birth of the first IVF baby (4, 5).

FET plays an increasingly important role in IVF cycle in women with a fresh embryo transfer does not result in pregnancy or in those who return with a second child. Due to decreased number of embryos per transfer and improvement in the laboratory techniques recently, the number of FET cycles to obtain pregnancy have been increased (6, 7). In addition, combined with clinical and literature reports, the incidence of vaginal bleeding in FET is high and has adverse effects on pregnancy outcomes (8). Besides, the pregnancy rate after FET cycles have been found to be lower than that after fresh embryo transfer (9). How to improve the OPR of FET and reduce the rate of early abortion has become an important research direction.

Traditional Chinese Medicine (TCM) is a kind of traditional treatment method with thousands of years history in China, and some previous studies have shown its unique experience in assisting pregnancy and reduce vaginal bleeding in early pregnancy (10, 11). Similar to the notion of “hypothalamus-pituitary-ovary axis” that is established by Western medicine, TCM also has deeply studied the reproductive regulation of kidney and proposed the concept of “kidney-Tian Gui -Chong Ren-uterine axis.” According to TCM, “Kidney Governs Reproduction,” and female infertility is closely related to kidney deficiency, and the main therapeutic principle of it involves tonification of the kidney.

GSATP is widely used as an adjunctive therapy in women with threatened abortion in China and the clinical effects reflected by the patients remained satisfactory (12). The function of GSATP is nourishing yin and tonifying the kidney, strengthening Chong and prevent miscarriage. GSATP is used in the early threatened abortion, which belongs to the kidney yin deficiency syndrome of traditional Chinese medicine. Gushen’antai pills (GSATP) are made up of “Dodder, uncaria, Scutellaria, Atractylodes macrocephala, white peony, rehmannia, Polygonum multiflorum, Dipsacus, Cistanche deserticola, mulberry parasitism.” The main components of GSATP include baicalin, Atractylodes macrocephala polysaccharide, flavonoids from Cuscuta chinensis, rhynchophylline, polysaccharides, Cistanche polysaccharides, stilbene glycosides and anthraquinone glycosides and triterpenoid saponins, etc. Modern pharmacological studies have found that these ingredients can improve vascular function, regulate immune activity, inhibit uterine contraction and improve ovarian endocrine function (13–16), so GSATP may play a role in promoting embryo implantation and preventing pregnancy. However, to improve the success rate of FET is a complex process and has not been fully studied. Therefore, in treating complex diseases, multi-targeted therapy such as TCM might have unique advantages over western medicine treatment alone. Although GSATP is associated with very good response in patients, lack of high-quality evidence-based medicine has restricted its promotion. The combination of evidence-based medicine, modern medicine and traditional Chinese medicine is a huge field that involves continuous attention and efforts.

This multi-center, double-blinded, randomized, placebo-controlled trial aims to investigate the effects of Chinese patent medicine GSATP on the OPR of HT FET cycles in women. This may provide a useful attempt for the clinical application of GSATP and the improvement of the clinical efficacy of HT cycle FET patients.

## Materials and Methods

### Patients

This multicenter, double-blind, randomized, placebo-controlled trial enrolled patients from the reproductive center of Affiliated Hospital of Shandong University of traditional Chinese Medicine, Maternity and Child Health Care of ZaoZhuang, Shanxi Maternal and Child Health Care Hospital, The First Affiliated Hospital of Wenzhou Medical University and Jinan Military General Hospital between November 2019 and May 2020 to investigate the safety and efficacy of GSATP. This study was conducted according to the Declaration of Helsinki and approved by the Reproductive Medicine Ethics Committee of the Affiliated Hospital of Shandong University of Traditional Chinese Medicine (approval number 20191109). Written informed consent was obtained from patients before study enrollment. The trial was registered on the Chinese Clinical Trial Registry (ChiCTR1900026737).

Patients were considered eligible if they met the following inclusion criteria: 1. between 22 and 40 years of age; 2. with two or more high-quality transplantable frozen fetuses; 3. received HT FET cycle; 4. received less than 3 previous FET cycles, with less than 2 unexplained abortions and less than 2 implant failures; 5. have not received similar drug treatment; and 6. with no history of mental illnesses, no abnormalities of liver and kidney function and electrocardiogram.

Exclusion criteria were as follows: patients with 1. BMI ≥30 kg/m^2^ (17); 2. abnormal development of reproductive system and one abnormal chromosome karyotype of both male and female; 3. major systemic diseases, such as rheumatoid and systemic lupus erythematosus; and 4. endometriosis, adenomyosis, or hydrosalpinx.

### Study Design

Subjects were randomly assigned to one of the two groups in a 1:1 ratio, in which one group received routine treatment of intramuscular (im) injection of progesterone (Tianjin Jinyao Amino Acid Co., Ltd., 1 ML: 10 mg) 10 mg qd and daily oral administration of didroxyprogesterone tablets 10mg bid (manufacturer: Abbott Biologi CALS B.V., Netherlands; Registration Certificate No.: h20130110; specification: 10 mg) plus GSATP (N  =  136), while the other group received routine treatment plus placebo (N  =  135). Patients, clinicians, researchers, and all other study personnel were blinded throughout the study.

Randomization was achieved by computer-generated random schedule in R software (version 3.5.1). Drugs are uniformly packed and distributed in the order of random distribution. Random coding is the unique identifier given to each patient when recruited. Each drug sample was allocated with an emergency signs as decode. A random key in duplicate was sealed in an envelope and given to the main participants in all the five participating clinical centers. At the end of the study, statistical analysts, researchers and staff with access to the random key will reveal the blindness. All patients received same dosage of GSATP or placebo, 6g, thrice a day (tid).

The study drug GSATP (Product specification: 6g* 9 bags) and placebo were provided and were relabeled by the Beijing bran Pharmaceutical Inc. Placebo is made up of a certain amount of starch and glucose, and is shaped like GSATP according to the national drug standards of the State Food and Drug Administration of China. On day 1 of endometrial transformation, a conventional oral dose of GSATP (6g tid) or a placebo (6g tid) was taken. Patients are required to complete their daily medication. Clinical pregnancy was confirmed by B-ultrasound 35 days after FET. Pregnant women should continuously take the previous medication by until week 12 of pregnancy and those who are not pregnant should stop taking the medication. Weeks of gestation were calculated using days of FET plus 18 days, regardless of whether the transfer was a D3 embryo or a blastocyst. Therefore, depending on the difference between the D3 embryo and the blastocyst, the duration of treatment is approximately 69 or 71 days.

### Endpoint

The primary endpoint was OPR at week 12 of pregnancy and the secondary endpoints were IR, CPR and AR. The OPR, IR, CPR and AR were measured at the end of the experiment. Hormone levels were monitored and recorded at FET on days 1, 7 and 14. Due to wide range of targets for TCM, potential beneficial or adverse reactions in patients should also be recorded during each follow-up visit.

Follow-up was performed in the outpatient department at weeks 2, 5, 7, 9 and 10 after FET. Patients were free to discontinue the experiment at any time. There was some variation in the follow-up, in which B-ultrasound measurements were recorded at each time point after confirming the pregnancy, but some measurements were recorded only at baseline such as the basic endocrine level and endometrial thickness on the day of transplantation.

Participants’ spontaneous and self-reported adverse events related to GSATP were recorded at each visit from baseline to week 12 of pregnancy by using the non-guided questioning method. Any symptom, syndrome or disease affecting the patient’s health and occurring during observation period, and any condition detected by other diagnostic procedures, such as unplanned measures that needs to be taken, leads to withdrawal from the study, or abnormal results found by laboratory examination were recorded. The standard classification of severity of adverse reactions were evaluated as follows: mild: the symptoms and signs can be detected by the patient, but can be tolerated without affecting the daily life activities of the patient and continue the pregnancy; moderate: uncomfortable and affects the patient’s diet, causing symptoms such as threatened abortion; and severe: affects the patient’s life and health, resulting in termination of pregnancy.

### Sample Size Calculation and Statistical Analysis

The sample size calculation was done based on previous studies, in which the efficacy of GSATP was 65%, α = 0.05 and β = 0.2. According to 1:1 ratio, there were 130 patients in the treatment group and 130 in the placebo group. Considering the complexity of clinical practice, the loss rate is estimated to be 0.1, eventually 300 patients were assessed and 29 patients were excluded due to non-compliance of the inclusion criteria. Finally, 271 patients were recruited (136:135 for GSATP and placebo), clinical data were available for all these patients. The data were analyzed for intentional treatment. All patients were randomly assigned before the first treatment without withdrawing and severe adverse events were included in the efficacy analysis. The study data were collected and managed by non-clinical staff who were responsible for data management in each clinical center. The data were shown as follows: continuous variables with normal distribution were presented as means ± SD, and the count data are presented in the form of n (%). Statistics were run using SPSS version 21 software (SPSS, Inc., Chicago, IL). The differences between the two groups were detected using χ^2^ for counting data or *t* test used for comparative analysis of measuring data. *P* values of less than 0.05 were considered to be statistically significant.

## Results

### Baseline Characteristics

**Figure 1:**
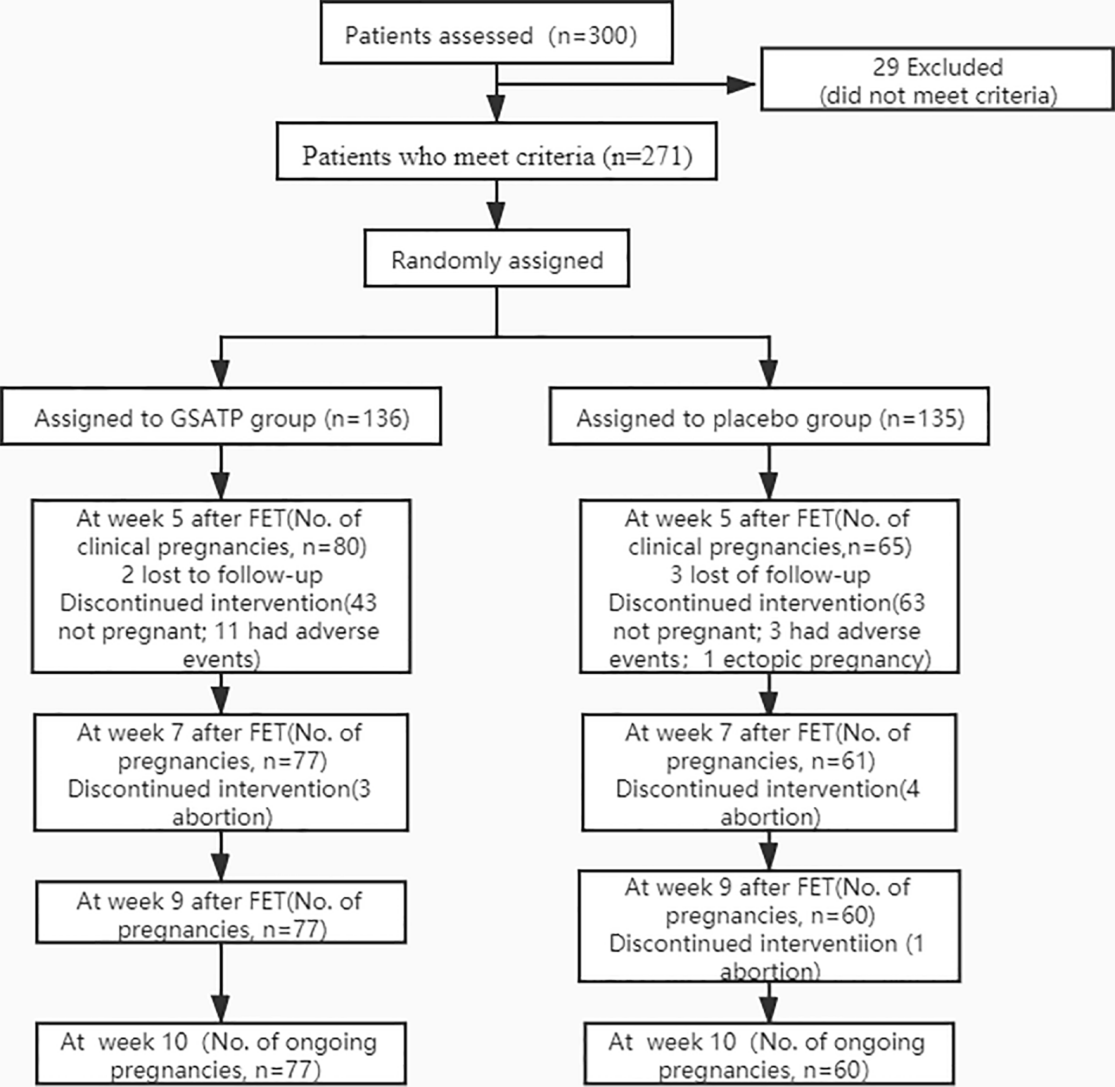
CONSORT diagram of participant randomization, treatment, and follow-up for ongoing pregnancy.

Of the 271 included patients, only 137 completed the study at 6-month follow-up visit (**Figure 1**).

A total of 300 HT FET cycle patients were screened from the 5 hospitals, with approximately an average of 60 patients per hospital participated in the study, and were randomly assigned to GSATP group and Placebo group in each hospital. After ruling out 29 cases who did not meet the inclusion criteria, a total of 271 patients were recruited. Of these, 136 patients received GSATP treatment and 135 patients received the same dose of placebo. The loss to follow-up rate was less than 2%. Patients discontinued the drug due to adverse events, which were more commonly seen in the GSATP group, and primarily included the gastrointestinal side effects. Also the baseline characteristics and clinical index on the day of FET were similar between the two groups (**Table 1**).

**Table 1:** Baseline characteristics.

Characteristics	GSATP (n = 136)	Placebo(n = 135)	*P* value
Mean (SD) age, years	32.34 (4.53)	31.74 (4.70)	0.689
Mean (SD) BMI, kg/m^2^	23.41 (3.76)	23.31 (3.65)	0.499
Mean (SD) no. of high quality embryos (n)	3.50 (1.49)	3.23 (1.34)	0.221
Mean (SD) previous FET times (n)	1.40 (0.74)	1.58 (0.87)	0.079
Mean (SD) duration of infertility, years	2.96 (1.91)	2.99 (1.84)	0.923
Mean (SD) outcome of the previous IVF cycle			
No. of retrieved oocytes (n)	15.65 (8.46)	14.87 (13.13)	0.565
No. of fertility (n)	9.90 (5.83)	9.28 (5.97)	0.616
Total Gn (U)	2,198.88 (1003.52)	2,236.00 (1024.86)	0.763
Mean (SD) Basal hormone levels			
FSH (IU/L)	7.35 (3.28)	7.17 (2.50)	0.178
LH (IU/L)	6.15 (4.84)	5.89 (5.21)	0.260
E2 (pg/ml)	52.40 (28.58)	48.58 (19.48)	0.199
Case (%), Infertility reason			
Tubal factor	95 (69.85)	92 (68.15)	0.762
Male factor	29 (21.32)	19 (14.07)	0.118
Unexplained	5 (3.68)	11 (8.15)	0.546
Both factor	7 (5.15)	13 (9.63)	0.158
Mean (SD) intimal thickness, mm	9.42 (1.86)	9.12 (1.12)	0.104
Mean (SD) average no. of FET embryo transfer (n)	1.88 (0.32)	1.82 (0.38)	0.172
Case (%) no. of day 3 embryo (n)	240 (93.75)	232 (94.31)	0.792
Case (%) no. Of Blastocyst (n)	16 (6.25)	14 (5.69)	0.792
Case (%) outcome of the previous FET cycle	GSATP (n=129)	Placebo(n=134)	*P* value
Clinic pregnancy rate	65 (50.39)	69 (51.49)	0.902
Abortion rate	8 (6.20)	7 (5.11)	0.795
Live birth rate	52 (40.31)	55 (41.04)	0.903

SD, standard deviation; BMI, body mass index; FET, frozen-thawed embryo transfer; No, number; LH, luteinizing Hormone; FSH, follicle stimulating hormone.

### Efficacy

#### Main Outcome

Implantation rate (%) = number of implantation gestational sac/number of transferred embryo×100%, clinical pregnancy rate (%) = number of clinical pregnancy/number of transfer cycle×100%, ongoing pregnancy rate (%) = number of continuous pregnancy/number of transfer cycle×100%, abortion rate (%) = number of abortion/number of clinical pregnancy×100%, vaginal bleeding or brown discharge rate = number of vaginal bleeding or brown discharge/number of clinical pregnancy×100%. The outcome was shown in **Table 2**, the data analysis shown in **Table 2** was began in FET week 5. The results showed that the OPR at week 12 of pregnancy was higher in the GSATP than placebo group (56.52% vs. 44.44%, *p* = 0.045), while vaginal bleeding or brown discharge rate was lower than that in the placebo group (10% vs. 23.08%, *p* = 0.032), showing statistically significant difference. However, there is no statistical difference in CPR (58.52% vs. 48.15%, *p* = 0.078), IR (35.16 vs. 27.64%, *p* = 0.07) and AR (3.75% vs. 7.69%, *p* = 0.504) between the two groups.

**Table 2:** The pregnancy outcome [Case (%)].

Index	GSATP (n=136)	Placebo (n=135)	*P* value
Clinic pregnancy rate	80/136 (58.82)	65/135 (48.15)	0.078
Implantation rate	90/256 (35.16)	68/246 (27.64)	0.070
Abortion rate	3/80 (3.75)	5/65 (7.69)	0.504
Vaginal bleeding or brown discharge rate	8/80 (10.00)	15/65 (23.08)	0.032
Ongoing pregnancy rate	77/136 (56.62)	60/135 (44.44)	0.045

#### Adverse Events

**Table 3** reported all adverse events that occurred during the study. Eleven patients (8.09%) reported adverse events in the GSATP group, and 3 patients (2.22%) reported adverse events in the placebo group. The adverse events reported by 11 patients in the GSATP group were thought to be associated with GSATP treatment. Adverse reactions to GSATP included gastrointestinal discomfort, diarrhea, allergies, and skin rash. All these 11 patients discontinued the study.

**Table 3:** Adverse events during the study period.

Variable	Patients, n (%)		*P* value
	GSATP (n = 136)	Placebo (n = 135)	
Adverse events			
Ectopic pregnancy	0	1 (0.74)	0.498
Allergies, skin rash	1 (0.74)	1 (0.74)	0.749
Diarrhea	2 (1.47)	0	0.251
Gastrointestinal discomfort	8 (5.88)	1 (0.74)	0.036
Total events	11 (8.09)	3 (2.22)	0.051

#### Subgroup Analysis

The subgroup analysis outcome was shown in **Table 4**. The results showed that there was no significant difference in the pregnancy outcomes between GSATP group and Placebo group when the 30-year-old was used as the dividing line. However, further age subgroup analysis showed that there were significant differences in CPR (74.19% vs. 54.17%, *p* = 0.004) and OPR (72.04% vs. 51.04%, *p* = 0.003) between GSATP group and Placebo group when the patient was younger than 35 years old. Besides, the vaginal bleeding or brown discharge rate was found higher (11.90% vs. 84.60%, *p <* 0.001) when age ≥ 35 years old between GSATP group and Placebo group.

**Table 4:** The pregnancy outcome of different ages [Case (%)].

Group	Age ≤30 years	Age >30 years	*P* value
GSATP/placebo	54/64	82/71	0.222
Index	Age ≤30		
	GSATP (n=54)	Placebo (n=64)	*P* value
Clinic pregnancy rate	38/54 (70.37)	38/64 (55.88)	0.250
Implantation rate	45/73 (61.64)	41/66 (62.12)	0.350
Abortion rate	1/38 (2.63)	2/38 (5.26)	0.500
Vaginal bleeding or brown discharge rate	2/38 (5.26)	3/38 (7.89)	0.500
Ongoing pregnancy rate	37/54 (68.52)	36/64 (56.25)	0.188
Index		Age > 30 years
	GSATP (n=82)	Placebo (n=71)	*P* value
Clinic pregnancy rate	42/82 (51.22)	27/71 (38.03)	0.107
Implantation rate	45/76 (59.21)	27/51 (52.94)	0.584
Abortion rate	2/42 (4.76)	3/27 (11.11)	0.373
Vaginal bleeding or brown discharge rate	5/42 (11.90)	13/27 (48.15)	0.002
Ongoing pregnancy rate	40/82 (48.78)	24/71 (33.80)	0.072
Group	age <35	age≥35	*P* value
GSATP/Placebo	93/96	43/39	0.692
Index		Age < 35
	GSATP (n = 93)	Placebo (n = 96)	*P* value
Clinic pregnancy rate	69/93 (74.19)	52/96 (54.17)	0.004
Implantation rate	78/130 (60.00)	41/66 (62.12)	0.774
Abortion rate	2/69 (2.90)	3/52 (5.77)	0.746
Vaginal bleeding or brown discharge rate	5/69 (7.25)	5/52 (9.62)	0.639
Ongoing pregnancy rate	67/93 (72.04)	49/96 (51.04)	0.003
Index	Age≥35		
	GSATP (n=43)	Placebo (n=49)	*P* value
Clinic pregnancy rate	11/43 (25.58)	13/39 (33.33)	0.441
Implantation rate	11/19 (57.89)	13/26 (50.00)	0.600
Abortion rate	1/11 (9.09)	2/13 (15.39)	0.643
Vaginal bleeding or brown discharge rate	5/42 (11.90)	11/13 (84.62)	<0.001
Ongoing pregnancy rate	10/43 (23.26)	11/39 (28.21)	0.608

GSATP use due to the principle of health. No severe adverse events were observed during this trial. The probability of side effects or adverse events of GSATP treatment was less, and the effect remained acceptable.

## Discussion

In this study, HT FET cycle patients were focused on to observe the efficacy and safety of GSATP. The results showed that GSATP improved the OPR and decreased vaginal bleeding or brown discharge rate of HT FET cycle patients while the AR, IR and CPR showed no significant differences between GSATP and placebo groups during the 6-month trial period. Subgroup analysis also showed that patients aged 30 to 35 years might be the most suitable beneficiaries. In addition, the safety of GSATP is relatively accepted due to the absence of major adverse reactions during the study period. These results suggest a relatively significant therapeutic effects of GSATP on HT FET cycle patients undergoing either early cleavage or blastocyst embryo transfer, at least during this 10-week course of treatment.

A significant difference was observed in the OPR between GSATP and placebo groups, and this was consistent with that of the previous research findings (4), what is more, our OPR (56.62% vs. 38.9%) was also higher than previous studies (18), however, this difference may be due to differences in inclusion criteria and sample size. Because the *p* value is only 0.045, which is on the edge of statistical difference, we are cautious about the results of our study and hope to expand the sample size in the future to obtain more accurate evidence-based medicine. However, no statistically significant difference was found in CPR, IR and AR between the two groups, which was not in line with that of the previous research findings (19–21). Notably, an upward trend of CPR and IR was observed in GSATP group when compared to placebo group, and this inconsistency might be due to different types of subjects selected and inclusion of small number of cases. Another interesting discovery is that vaginal bleeding or brown discharge rate in GSATP group was lower than that in placebo group. Bleeding in early pregnancy might be associated with endometrial implantation, and in severe cases it might result in miscarriage (22). This might be due to the drug components included in GSATP, which could increase the response of hypothalamus-pituitary-ovary axis to reproductive hormones, and regulate the hormone levels in the body, especially the estrogen-like effect or improve the serum estrogen concentration, improving the conditions of embryo implantation (14, 23–25). At present, there are few studies on this topic, and further research is warranted to verify the specific underlying mechanism.

However, this study has some limitations. Firstly, age is the most important factor in the outcome of FETs, our age inclusion criteria is based on previous similar studies (20). However, most of the patients selected in this study were non-elderly patients who were in good general condition, and this might exaggerate our results. Secondly, from a clinical point of view, the good outcomes of pregnancy is much important, for safety reason as well, however the survival rate of FET was not observed in this study because of short observation period. Thirdly, it is well known that TCM should be treated according to the syndrome differentiation to achieve its maximum effect. In our experiment, patients were not treated according to syndrome differentiation, and this is because some studies included pregnant women (about 80%) with kidney deficiency (26), which might in turn impact our experimental results. Last but not the least, luteal support in our study was based on the adjustment of clinical doctors and the principle of benefiting patients, which is not completely consistent. However, as the basic situation of GSATP and placebo groups basically remained the same, the flexible adjustment of their individuals within the regular range showed no effect on our final results.

Luteal support is currently the most commonly used treatment to increase the odds of pregnancy in FET patients, but there is no agreement on the best formulation, approach, dose or duration of progesterone use, and how to improve the clinical effects of FET, and these are the problems to be resolved (27). TCM has been used for centuries to prevent and treat diseases and its effectiveness has been proven in clinical practice. The multitarget effects of TCM therapy might also have great advantage in treating infertility as an adjuvant therapy (28, 29).

This multi-center, randomized, double-blinded, placebo-controlled clinical study showed for the first evidence that GSATP, as a relatively safe and effective treatment, may have potential to improve the OPR and decrease vaginal bleeding or brown discharge rate in HT FET cycle patients.

## Data Availability Statement

The raw data supporting the conclusions of this article will be made available by the authors, without undue reservation.

## Ethics Statement

The studies involving human participants were reviewed and approved by Reproductive Medicine Ethics Committee of the Affiliated Hospital of Shandong University of Traditional Chinese Medicine (approval number 20191109). The patients/participants provided their written informed consent to participate in this study.

## Author Contributions

X-lC wrote this manuscript. J-yS revised the manuscript. X-xZ, Y-hC, Y-lT, and H-pL participated in the data processing stage. T-yD made and provided placebo for the experiment. Z-gS designed the study, drafted and revised the manuscript.

## Funding

This study is supported by the National Natural Science Foundation of China (NSFC: 81874484).

## Conflict of Interest

The authors declare that the research was conducted in the absence of any commercial or financial relationships that could be construed as a potential conflict of interest.
